# Findings from Integrated Behavioral and Biologic Survey among Males Who Inject Drugs (MWID) — Vietnam, 2009–2010: Evidence of the Need for an Integrated Response to HIV, Hepatitis B Virus, and Hepatitis C Virus

**DOI:** 10.1371/journal.pone.0118304

**Published:** 2015-02-18

**Authors:** Patrick Nadol, Siobhan O’connor, Hao Duong, Linh-Vi N. Le, Pham Hong Thang, Tran Hong Tram, Hoang Thi Thanh Ha, Michelle S. Mcconnell, Jeff Partridge, John Kaldor, Matthew Law, Tuan Anh Nguyen

**Affiliations:** 1 U.S. Centers for Disease Control and Prevention, Division of Global HIV/AIDS, Hanoi, Vietnam; 2 Kirby Institute for Infection and Immunity, University of New South Wales, Sydney, Australia; 3 U.S. Centers for Disease Control and Prevention, Division of Viral Hepatitis, Atlanta, Georgia, United States of America; 4 National Institute of Hygiene and Epidemiology, Hanoi, Vietnam; 5 U.S. Centers for Disease Control and Prevention, Division of Influenza Control, Atlanta, Georgia, United States of America; University of the Witwatersrand, SOUTH AFRICA

## Abstract

**Introduction:**

Given the overlapping modes of transmission of HIV, hepatitis B virus (HBV), and hepatitis C virus (HCV), understanding the burden and relationship of these infections is critical for an effective response. Representative data on these infections among males who inject drugs (MWID), the key high-risk population for HIV in Vietnam, are currently lacking.

**Methods:**

Data and stored specimens from Vietnam’s 2009-2010 Integrated Biologic and Behavioral Survey, a cross-sectional study among high-risk populations, were used for this analysis. Plasma samples were tested for HIV, HBV, and HCV using commercial assays. A questionnaire was administered to provide demographic, behavior, and service-uptake information. Provincial-level analyses were conducted to profile MWID enrollees and to provide estimates on the prevalence of HIV, HBV, and HCV infection.

**Results:**

Among 3010 MWID sampled across 10 provinces, the median (range) HIV prevalence was 28.1% (1.0%-55.5%). Median prevalence for current HBV infection (HBsAg+) was 14.1% (11.7%-28.0%), for previous exposure to HBV (total anti-HBc+) was 71.4% (49.9%-83.1%), and for current or past HCV infection (HCV Ag/Ab+) was 53.8% (10.9%-80.8%). In adjusted analysis, HBsAg+ (aOR: 2.09, 1.01-4.34) and HCV Ag/Ab+ (aOR: 19.58, 13.07-29.33) status were significantly associated with HIV infection; the association with total anti-HBc+ approached significance (aOR: 1.29, 0.99-1.68).

**Conclusion:**

The prevalence and association between HIV, HBV, and HCV are high among MWID in Vietnam. These findings indicate the need for integrated policies and practice that for the surveillance, prevention, screening, and treatment of both HIV and viral hepatitis among MWID in Vietnam.

## Introduction

Similar modes of transmission of human immunodeficiency virus (HIV), hepatitis B virus (HBV) and hepatitis C virus (HCV) can lead to an increased risk of HIV co-infection with HBV or HCV.[1] Because HIV, HBV, and HCV can be efficiently transmitted via percutaneous exposure to blood, people who inject drugs (PWID) are at especially high risk for infection and co-infection with these viruses and for transmission to others through unsafe needle sharing or sex practices.[2]

Worldwide, of an estimated 240 million chronic HBV infections, 1.2 million (0.5%) occur among people who inject drugs (PWID); of 170 million chronic HCV infections, 10 million (5.9%) occur among PWID.[3] Chronic HBV and HCV infections are associated with increased risk of cirrhosis and liver cancer and are responsible for more than 1 million deaths annually.[3–6] Recent estimates also indicate that a significant amount of hepatitis B, hepatitis C, and HIV burden, as determined by disease specific disability-adjusted life years, can be attributed to illegal injection drug use.[7] The prevalence of HCV co-infection among HIV-infected PWID (HCV/HIV) often exceed 50%.[1,2,8–11] Among people infected with HIV, co-infection with viral hepatitis adversely impacts morbidity and mortality and is becoming a leading cause of death among co-infected PWID, even in the era of anti-retroviral therapy (ART) to treat HIV/AIDS.[12,13]

Up to 335,000 active males who inject drugs (MWID) live in Vietnam, making it one of the six highest burden countries for injecting drug use worldwide.[14,15] Vietnams HIV epidemic is concentrated among high-risk populations, specifically MWID, female sex workers (FSW), and men who have sex with men (MSM).[15] Although these populations are not mutually exclusive, MWID are reported to have the highest HIV burden with national prevalence estimated to be 14.4%MWID [15] The prevalence of current hepatitis B surface antigen (HBsAg), a marker of active HBV infection and infectiousness, varies between 5.7% and 24.7% by population. An estimated 8.4 million (~10% of the population) people in Vietnam are also chronically infected with HBV.[16–18] The prevalence of antibody to HCV (anti-HCV), indicative of current infection or previous exposure to hepatitis C, ranges between 0.38% and 1.7% in the general population in Vietnam but between 31–98.5% among MWID.[2,16]

These hepatitis data come from a variety of relatively small, localized surveys which lack the ability to provide nationally or provincially representative data regarding the epidemiology and frequency of HBV, HCV, or co-infection with HIV for Vietnam. Neither HBV nor HCV is a required reportable disease in Vietnam, and the country currently lacks routine hepatitis surveillance systems.[16,17] Such data are needed about HBV and HCV infection among key populations, such as MWID, for understanding the burden and profile of these key infections to organize appropriate resources for an effective response. The objectives of this analysis are to profile behaviors relevant for HIV, HBV, and HCV acquisition and transmission; to present representative estimates of HIV, HBV, HCV, and co-infection prevalence; and to identify risk factors associated with HIV infection among MWID in Vietnam.

## Methods

### Study Population and Sampling

Data from the 2009 Integrated Behavioral and Biologic Survey (IBBS) were used for this study. The 2009 IBBS was a cross-sectional survey of populations at increased risk for HIV in Vietnam including male injecting drug users (MWIDs). The main objectives of IBBS were to assess a representative sample of these key populations to determine HIV prevalence, associated behaviors (e.g. condom use and needle-sharing behaviors), and exposure to HIV prevention and care services. During September 2009–February 2010, MWID, defined as individuals self-reporting illicit injecting drug use at least one time in the previous 30 days aged ≥18 years, were sampled across 10 geographically diverse provinces in Vietnam that have received focused support in addressing HIV via the US President’s Emergency Plan for AIDS Relief (PEPFAR) program. MWID were sampled in each province using either respondent driven sampling (RDS) (Hanoi, Da Nang, Ho Chi Minh City (HCMC), Can Tho) or two-staged time-location sampling (TLS) (Hai Phong, Quang Ninh, Nghe An, Yen Bai, Dong Nai, An Giang) based on formative provincial mapping conducted by the study team as described in previous reports.[19]Inclusion of injecting drug users in the Vietnam IBBS is limited to males given the assumption that the vast majority of drug injectors are male and to allow comparability to previous surveys.

Following recruit eligibility screening and informed consent, trained staff conducted individual interviews using a structured questionnaire. Blood samples were collected for HIV testing and remnant samples were stored for potential testing in the future. Participants were provided coupons and encouraged to return to obtain the HIV test results. All participants were compensated between 50,000–100,000 VND ($2.50-$5.00) for their time and travel expenses. A monetary compensation was also provided to RDS recruits who subsequently recruited peers into the study at defined study locations.

Written, informed consent was obtained by reading a standardized study description and consent form that was also provided to the study recruit for their review and signed agreement. This consent process, which included the provision for future testing on stored specimens, was approved by the below-described ethics review boards.

### Ethical approval

The study protocol was reviewed and approved by the Vietnam National Institute of Hygiene and Epidemiology (NIHE) Ethics Review Board and the Internal Review Board of the U.S. Centers for Disease Control and Prevention.

### Laboratory analysis

HIV status was determined per national guidelines by screening for antibody to HIV using Genscreen HIV Ultra HIV Ag/Ab (Bio-Rad) with confirmatory testing of those screened as HIV-positive by Determine HIV ½ (Alere) and Murex HIV Ag/Ab Combination (DiaSorin). An enrollee was classified as HIV-positive if all three tests were positive for HIV-infection. Ten percent of HIV-negative samples and 5% of HIV-positive samples were randomly selected and re-tested for quality assurance at the National Reference Laboratory at NIHE in Hanoi.[19]

In August 2012, stored plasma from the IBBS study underwent serologic testing for HBV and HCV infection at the Vietnam National Institute of Hygiene and Epidemiology (NIHE) in Hanoi. Testing for HBsAg to determine current HBV infection was conducted by using the Murex HBsAg Version 3; an optical density (OD) value >0.05 over the mean negative control was interpreted as positive for HBsAg.[20] Specimens with indeterminate, weakly positive or negative HBsAg results were also tested for total anti-HBc to determine previous HBV infection by using the ETI-AB-COREK PLUS (DiaSorin). The combination of results was used to classify the HBV infection status of each subject as: ‘current or active HBV infection’ (HBsAg+), ‘prior HBV infection’ (HBsAg-/total anti-HBc+) or ‘susceptible to future HBV’ (HBsAg-/total anti-HBc-). Specimen quantity was insufficient to test for antibody to hepatitis B surface antigen (anti-HBs). As such, a small proportion of MWID classified as susceptible might have been immune from vaccination although we anticipate this to be a small percentage given the low coverage rates of HBV vaccination in this age group and population.[16]

Stored specimens also underwent combined testing for the presence of antibody to HCV or HCV antigen (Murex HCV Ag/Ab Combination, DiaSorin). Specimens that tested indeterminate or weakly positive were re-tested by the same methods. An OD value > 0.20 over the mean negative controls was used as the cut-off definition for a positive result.[21] The subject’s HCV infection status was classified as: ‘past or current HCV infection’ (HCV Ag/Ab+); ‘indeterminate/weakly positive’ (if repeat testing remained indeterminate/weakly positive); or ‘no evidence of HCV infection’ (HCV Ag/Ab-).

### Statistical Analysis

Simple frequencies and proportions were calculated for categorical variables by province; and mean and medians were calculated for continuous variables. Prevalence estimates for HIV, HBV, HCV, and co-infections among MWID with 95% confidence intervals (95% CI) were calculated by province. Comparison of categorical data was done using the chi-square test or Fisher’s exact test (if expected frequencies were less than 5). Any indicator missing more than 5% of the total responses by province were reported in the respective output tables. For provinces sampled through RDS, analyses were done by using RDS Analyst (v0.1)[22] with successive sampling estimator [23] applied apriori using mid-range MWID provincial population size estimates approved by the Vietnamese Ministry of Health. [24–25] The remaining six provinces were analyzed using STATA (v.12.0).[26]

Median values and ranges were calculated for the total of provinces sampled. The Mann-Whitney test was used to compare median values for different groups. Pairwise correlations, and their significance levels using t-test probabilities, were calculated and reported to estimate the provincial-level variation between HIV prevalence and HBV and HCV prevalence. For univariate and multivariate risk factor analysis, unweighted odds ratios (OR) and 95% CI were calculated by stratified (conditional) logistic regression with HIV status as the outcome variable and stratified by ‘province’ to protect against any confounding (e.g. Simpson’s paradox) effect that may appear in an unstratified analysis.[27] The rationale for combining data across all provinces was to provide an increased sample size effectively increasing the power to detect key estimates and associations resulting in more robust inferences. All independent variables indicating an association with HIV status (p< = 0.20) in the univariate analysis were entered into a multivariate conditional logistic regression model using backward step-wise selection and the Wald test after estimation to identify the most parsimonious model. Collinearity was assessed by examining the variance inflation factors (VIF) of the model; variables indicating strong collinearity (i.e. VIF >8.0) were removed from the final analysis.

## Results

### Population characteristics and risk behaviors

Among the 10 provinces, [28] 3010 (3%) of an estimated 98,900 [24] MWID were sampled. The median (range, by province) population was 2.03 (0.76–7.68) million (Table 1.1). The median estimated MWID population across the provinces sampled was 4,520 (1,197–46,213).[24,28] The median age was 30.0 (21.5–35.5) years with the majority (>50%) of enrollees self-reported never being married, but most (>90%) being sexually active.

**Table 1.1:** Demographic characteristics of MWID in select provinces in Vietnam, 2009.

Province		Hanoi*	Hai Phong	Quang Ninh	Nghe An	Yen Bai	Da Nang*	Dong Nai	HCMC*	Can Tho*	An Giang	Median (Range)
**Province Population**		6,844,100	1,904,100	1,177,200	2,952,000	764,400	973,800	2,720,800	7,681,700	1,214,100	2,153,700	2,028,900 (764,400–7,681,700)
**Estimated PWID population size**		46,213	7,194	4,873	7,922	4,166	1,197	2,127	21,566	2,263	1,379	4,520 (1,197–46,213)
**Sampled male MWID (n)**		297	298	299	298	348	288	300	310	272	300	298.5 (272–348)
**Age (years)**	Mean (sd)	31.5 (8.1)	35.8 (7.0)	31.6 (5.7))	30.4 (9.3)	34.6 (7.7)	24.9 (8.7)	28.1 (9.1)	29.2 (7.7)	32.2 (9.4)	25.6 (7.3)	31.0 (24.9–35.8)
	Median (IQR)	30.6 (25.7–35.6)	35.5 (31.2–40.4)	31.2 (27.5–35.2)	30.0 (24.6–35.0)	34.3 (29.2–39.3)	21.5 (19.4–26.2)	26.3 (21.0–31.4)	27.5 (24.0–33.0)	29.9 (26.0–38.3)	23.9 (20.0–28.4)	30.0 (21.5–35.5)
**Ethnicity**	Kinh (%)	99.3	100	98.7	99.3	82.3	100	98	97.4	97.4	97.7	98.35 (82.3–100)
**Education (%)**	Illiterate	3	0	0.3	0.3	2.9	0	3.7	5	12	14.1	2.95 (0–14.1)
	Primary	3.8	10.2	0.7	4.4	10.1	6.5	12.7	34.6	31.1	41.1	10.15 (0.7–41.1)
	Secondary or High School	89.6	88.2	96	82.2	81.4	87.2	78.9	56.9	55.4	44.8	81.8 (44.8–96)
	College/University	3.6	1.7	3	13.1	5.23	6.6	4.7	3.5	1.5	0	3.55 (0–13.1)
	Missing (#)	55	1	1	-	4	2	1	45	-	1	
**Marital status (%)**	Never Married	58.6	40.1	68.2	59.4	36	81.9	70	69.9	51.5	66.9	63.15 (36–81.9)
	Currently Married	30	24.2	21.4	35.2	47.6	12.5	23	16.5	28.3	20.7	23.6 (12.5–47.6)
	Divorced/Separated or Widowed	11.8	35.7	10.3	5.4	16.5	5.5	7.1	13.6	20.2	12.4	11.45 (5.4–35.7)
**Sexually active (i.e. ever had sex)**		92.6	96.3	80.9	95.3	97.1	90.1	77.3	93.2	95.3	82.3	92.9 (77.3–97.1)
Household Income (VND * 10,000)	Median (IQR) (VND*10,000)	200 (190)	180 (150)	250 (120)	150 (150)	150 (110)	100 (185)	150 (150)	150 (150)	200 (150)	150 (110)	150 (100–250)
**Unemployed (%)**		0.0	0.3	0.3	9.7	23.2	0.0	1.0	0.0	1.5	0.0	0.3 (0–23.2)
**Table 1.2: Risk behaviors among MWID in select provinces in Vietnam, 2009**												
**Average duration (years) of drug use (sd)**		9.5	11.30	8.7	6.1	9.4	4.7	6.6	7.6	8.8	5.6	8.2 (4.7–11.3)
**Average duration (years) of injection drug use (sd)**		6	7.4	7.2	4.4	6.8	3.6	5.7	5.4	6.3	4.7	5.9 (3.6–7.4)
**Average age at initiating drug use (sd)**		22.0 (6.6)	24.5 (6.6)	22.8 (5.1)	23.9 (6.2)	25.2 (6.5)	20.1 (6.4)	21.2 (6.0)	21.6 (7.1)	24.9 (12.8)	20.0 (5.9)	22.4 (20–25.2)
**Average age at initiative injection drug use (sd)**		25.6 (7.4)	28.5 (7.2)	24.3 (5.4)	25.7 (6.7)	27.7 (7.4)	21.3 (6.5)	22.1 (6.4)	23.7 (7.4)	27.6 (9.2)	20.9 (6.3)	25.0 (20.9–28.5)
**In past month how often have you injected drugs (%)**	> = 4 times/day	3.7	10.1	0.3	3	1.4	1	0	4.8	0.7	0.67	1.2 (0–10.1)
	1–3 times/day	86.4	89.6	93	64.9	55.3	67.7	47.7	94.8	86.3	83	84.65 (47.7–94.8)
	< 1 time/day	9.8	0.3	6.7	32.2	42.7	31.3	48	0.4	12.9	16.4	14.65 (0.3–48)
	Don’t know/no response	0	0	0	0	0.6	0	4.3	0	0	0	0 (0–4.3)
**Ever shared needles/syringes with others during injection drug use (95% CI)**		37 (29.5, 45.9)	31.2 (25.9, 36.5)	70.2 (65.0, 75.4)	56.4 50.7, 62.0)	57.6 (52.4, 62.8)	43.6 (35.5, 50.9)	36.7 (31.2, 42.1)	41.2 (32.8, 50.9)	36.6 (29.3, 43.9)	28.1	39.1 (28.1–70.2)
	Missing (#)	52	-	-	-	-	2	-			1	
**Frequency of needle/syringe sharing in the past 6 months (%)**	Always or Most of the time	2.7	0.3	0.3	1.3	0.6	4.9	3.3	3.2	5.9	4.7	3.0 (0.3–5.9)
	Occasionally	22.2	7.1	23.4	26.9	24.1	31.9	23.7	21.3	11	10.7	22.8 (7.1–31.9)
	Never	22.6	23.5	46.5	28.2	32.8	6.3	9	20.3	25.7	13	23.1 (6.3–46.5)
	No response	54.6	69.1	29.8	43.6	42.5	56.9	64	55.2	57.4	71.7	56.05 (29.8–71.7)
**Received free clean needles and syringes in the past 12 months % (95% CI, n)**		83.3 (71.6, 94.9), n = 80	86.6 (81.0, 92.2), n = 142	97.7 (95.8, 99.7), n = 220	94.3 (90.1, 98.4), n = 122	98.3 (96.6, 100), n = 233	98.4 (96.9, 99.9), n = 288	96.1 (92.7, 99.5), n = 127	90.3 (85.4, 95.1), n = 308	97.2 (94.4, 99,9), n = 141	98.4 (96.9, 100), n = 254	97.0 (83.3–98.4)
**Median (IQR) IDU network size**		12 (7–20)	9 (5–17)	12 (7.5–19.5)	7 (5–20)	10 (7–20)	7 (4–12)	6 (3–15)	10 (6–20)	5 (3–10)	8 (4–20)	8.5 (5–12)
**Median (IQR) number of different sexual partners in previous 12 months**		2 (1–4)	0 (0–1)	1 (0–1)	1 (1–4)	1 (1–2)	3 (1–4)	1 (1–2)	1 (0,1)	1 (0–1)	1 (1–3)	1 (0–3)
	Missing (#)	-	13	72	18	13	-	83	1	-	53	
**Proportion (%) reporting sexual relations with a female sex worker in the past 12 months**		45.6	33.3	23.1	47.9	35	42.8	15	17	20.7	31.9	32.6 (15–47.9)
	Missing (#)	80	169	195	56	71	-	120	-	-	109	
**Condom used with last sex with female sex worker (%, 95% CI, n)**		70.4 (58.2, 82.6), n = 143	91.1 (82.7, 99.5), n = 45	84.8 (72.4, 97.3), n = 33	81.5 (74.5, 88.5), n = 119	83.8 (76.5, 91.1), n = 99	77.7 (66.6, 88.8), n = 133	70.3 (55.3, 85.2), n = 37	49.1 (28.8, 69.5), n = 58	67.7 (51.6, 83.7), n = 65	81.7 (71.8, 91.6), n = 60	79.6 (49.1–91.1)
**Ever tested for HIV % (95% CI)**		34.9 (29.2, 40.6)	58.5 (52.7, 64.4)	68.4 (63.0, 73.8)	53.4 (47.7, 59.1)	38.9 (33.7, 44.1)	25.3 (20.1, 30.4)	25.4 (20.2, 30.6)	26.5 (21.5, 31.4)	36.8 (30.9, 42.8)	41.2 (35.2, 47.2)	38.0 (25.3–68.4)
**Ever been in a drug rehabilitation (06) center?**		58.2 (50.5, 65.9)	62.8 (57.3, 68.3)	67.2 (61.9, 72.6)	84.2 (80.1, 88.4)	61.1 (56.0, 66.2)	68.8 (63.4, 74.1)	18.7 (14.3, 23.2)	64.4 (59.1, 69.8)	53.7 (47.7, 59.6)	30.1 (24.9, 35.3)	62.0 (18.7–84.2)

*enrollees sampled using Respondent-driven sampling (RDS)

Key risk behaviors by province for HIV among those sampled are presented in Table 1.2. The median (range, by province) duration of drug use and injecting drug use was 8.2 (4.7–11.3) and 5.9 (3.6–7.4) years respectively. In all provinces except Dong Nai, the majority of enrollees reported injecting drugs one or more times per day with a median of 39.1% (28.1%-70.2%) of MWID reported having ever shared needles or syringes with others during injection drug use. Relatively few (3.0% [0.3%-5.9%]) MWID reported sharing needles ‘always or most of the time’ when they inject drugs, although 56.1% (29.8%-71.7%) of enrollees gave no response for this indicator. The vast majority (97.0% [83.3%-98.4%]) of all MWID sampled did report receiving free clean needles and syringes sometime in the past 12 months. An estimated 32.6% (15.0%-47.9%) of MWID reported having sexual relations with a female sex worker in the previous 12 months, and, of those who had, the majority (79.6% [49.1%-91.1%]) reported having used a condom during the last such encounter. A median of 38% (25.3–68.4%) of MWID reported previously testing for HIV.

### HIV, HBV, and HCV prevalence

**Fig 1:**
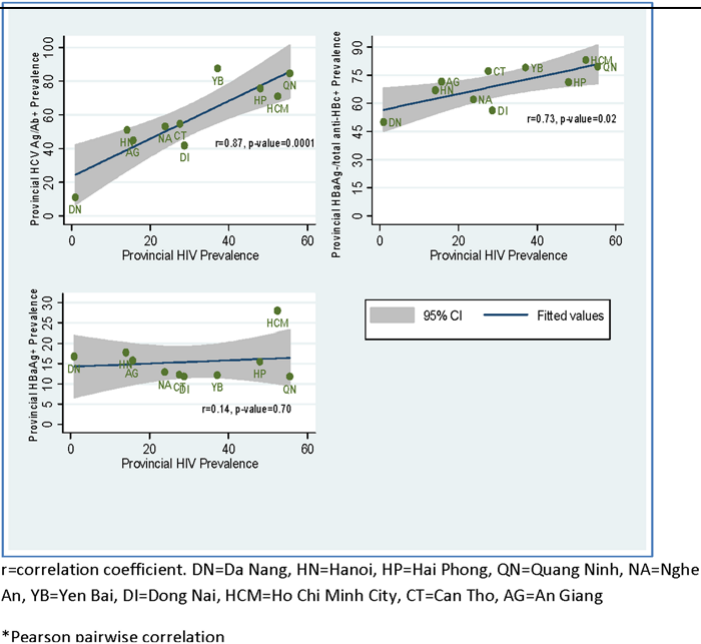
Provincial level correlation* of HIV to HBsAg, total anti-HBc, and HCV in the 2009 IBBS.

HIV prevalence among MWID varied widely by province (Fig. 1); the median HIV prevalence was 28.2% (1–55.5%) (Table 2). Across all 10 provinces, 14.1% (11.7%-28.0%) of MWID demonstrated active HBV infection (i.e., HBsAg+) and among those who were HBsAg-, 71.4% (49.9%-83.1%) had evidence of previous HBV infection (61.3% of the overall population sampled). Across provinces, median prevalence of past or current HCV infection among MWID overall was 53.8% (10.9%-87.4%). See the S1 Table for individual provincial-level data related to HIV, HBV, and HCV.

**Table 2:** HIV, hepatitis B virus (HBV), hepatitis C virus (HCV), and co-infection prevalence.

**Sero-marker**	**Median (Range) across sampled provinces% (95% CI)**
HIV	28.1 (1–55.5)
HBsAg+	14.1 (11.7–28)
HIV+	15.7 (9.6–66.7)
HIV-	16.4 (10.3–22.8)
HBsAg-/total anti-HBc+	71.4 (49.9–83.1)
HIV+	81.2 (31.7–100)*
HIV-	64.7 (49–78.1) *
HCV Ag/Ab+	53.8 (10.9–87.4)
HIV+	95.8 (35.5–99.4) *
HIV-	52.2 (10.9–80.8) *
**HBV, HCV and HIV prevalence (among all enrollees with a recorded result)**	
HBsAg+/HIV+ % (95% CI)	4.6 (0.9–13.4)
anti-HBc+/HIV+ % (95% CI)	19.7 (0.1–39)
HCV Ag/Ab+/HIV+ % (95% CI)	26.3 (14.1–55.2)
HBsAg+/HCV Ag/Ab+/HIV+ % (95% CI)	1.2 (0.3–2.9)

* indicate p-value <0.05

HIV-positive participants were more likely than HIV-negative participants to have evidence of previous HBV infection (81.2% vs. 64.7%, p-value<0.005); active HBV infection status did not differ significantly between HIV-positive and HIV-negative MWID (15.7% vs. 16.4%, p-value>0.05). Thus, a median (range) of 4.6% (0.9%-13.4%) of all MWID sampled were HIV-infected and currently co-infected with HBV, and 19.7% (0.1%-39.0%) were HIV-positive and evidenced past HBV infection (Table 2). In all provinces except Hanoi and Da Nang, past or current HCV infection was higher among HIV-positive than HIV-negative respondents (overall: 95.8% vs. 52.2%, p-value<0.0005) (Table 2). Overall, across all provinces, a median of 26.8% (14.1%-55.2%) of sampled MWID tested positive for both past or current HCV infection and HIV, with markedly high prevalence (> = 50%) among MWID in Hai Phong and Quang Ninh. In contrast, no participants in Da Nang province, with its relatively low HIV prevalence (1% [0%-2.4%]), tested positive for both HIV and past or current HCV infection, potentially indicating evidence of low HCV/HIV co-infection prevalence there (See supplemental table).

At the provincial level, HIV prevalence was significantly correlated with past HBV infection prevalence (r = 0.73, p-value = 0.02) and past or current HCV infection prevalence (r = 0.87, p-value = 0.0001) and weakly correlated with active HBV infection prevalence (r = 0.14, p-value = 0.70) (Fig. 1).

### Factors associated with HIV status

Bivariate analysis (Table 3a) at the individual level also identified associations between HIV infection and past HBV infection (OR: 2.40, 1.93–2.99) and past or current HCV infection (OR: 29.84, 20.64–43.16). The association between HIV and active HBV infection (OR: 1.19, 0.94–1.49) was statistically not-significant. Several other factors were also associated with HIV status, including: increased duration and frequency of injecting drug use and needle sharing, injecting heroin exclusively compared to also injecting other drugs, increased MWID network size, ever being tested for HIV infection and ever being detained in a drug rehabilitation center.

**Table 3:** Crude and Adjusted Associations between HIV prevalence (outcome) and select factors among MWID in Vietnam.

**Independent predictor (n, %)**	**Table 3a: Crude Associations between HIV prevalence and select factors**				**Table 3b: Adjusted Associations between HIV prevalence and select factors**		
	**Observations**	**Odds Ratio (OR)**	**95% Confidence Interval**	**p-value**	**Adjusted Odds Ratio (aOR)**	**95% Confidence Interval**	**p-value**
**Age Category**	2986 (% HIV+)						
18–24	881 (11.80)	1	-	-	1		
24–30	706 (41.08)	3.85	2.94, 5.03	<0.0005	1.85	1.30, 2.62	0.001
> = 30	1399 (38.03)	3.09	2.39, 3.98	<0.0005	1.31	0.93, 1.83	0.12
**Ethnicity Category**	3007						
Kinh	2909 (30.90)	1	-	-			
Other	98 (32.65)	0.87	0.55, 1.37	0.55			
**Education**	2982						
Illiterate	120 (19.17)	1	-	-			
Primary	445 (31.91)	1.82	1.09, 3.04	0.02			
Secondary	1198 (32.30)	1.85	1.13, 3.02	0.01			
High School	1087 (31.37)	1.46	0.88, 2.42	0.14			
College, University	132 (22.73)	1.34	0.72, 2.56	0.38			
**Marital Status**	3005						
Never Married	1800 (28.56)	1	-	-			
Currently Married	789 (32.07)	1.07	0.88, 1.31	0.48			
Divorced	286 (36.01)	1.15	0.87, 1.53	0.32			
Separated	110 (43.64)	1.36	0.90, 2.05	0.15			
Widowed	12 (60.00)	2.79	1.09, 7.15	0.03			
**Unemployed**	3000						
Yes	118 (44.92)	1	-	-			
No	2882 (30.46)	0.55	0.36, 0.83	0.004			
**Duration of drug use**	2952						
< 1 year	202 (4.46)	1	-	-			
1–3 years	535 (9.35)	2.3	1.10, 4.82	0.03			
3–5 years	383 (21.15)	5.17	2.50, 10.67	<0.0005			
> = 5 years	1832 (42.41)	11.66	5.87, 23.18	<0.0005			
**Duration of injecting drug use (IDU)**	2952						
< 1 year	391 (7.93)	1	-	-	1		
1–3 years	671 (13.71)	1.77	1.14, 2.74	0.01	1.13	0.66, 1.94	0.46
3–5 years	458 (28.38)	3.59	2.33, 5.54	<0.0005	1.74	1.04, 2.93	0.04
> = 5 years	1415 (46.64)	7.53	5.08, 11.19	<0.0005	3.03	1.87, 4.89	<0.0005
**Frequency of IDU (in the past month)**	3007						
> = 4 times per day	95 (28.42)	1	-	-			
2–3 times per day	1471 (33.99)	1.53	0.94, 2.47	0.09			
1 time per day	832 (26.20)	1.46	0.88, 2.44	0.15			
<1 time per day	592 (30.07)	2.17	1.28, 3.69	0.004			
Don’t know/no response	17 (47.06)	4.07	1.36, 12.11	0.01			
**Type of drugs injected in the past month**	3009						
Heroin exclusively	2751 (32.35)	1	-	-	1	-	-
Heroin as well as other drugs	175 (18.29)	1	0.64, 1.57	0.99	1.36	0.76, 2.43	0.31
Drugs other than heroin	83 (12.05)	0.41	0.20, 0.81	0.01	0.24	0.08, 0.68	0.01
**Frequency of needle/syringe sharing**	3007						
Never	1627 (22.56)	1	-	-			
Ever shared in the past year	977 (46.98)	2.87	2.38, 3.47	<0.0005			
Ever shared in the past month	403 (26.05)	1.47	1.13, 1.93	0.01			
**MWID network size**	2955						
<5	574 (23.87)	1	-	-			
5-<15	1318 (29.59)	1.18	0.93, 1.51	0.17			
>15	907 (35.75)	1.47	1.15, 1.88	0.002			
**Sexually active**	3009						
Yes	2711 (31.02)	1	-	-			
No	298 (30.54)	0.98	0.74, 1.30	0.90			
**Sex with female sex worker in past 12 months**	1947						
No	1216 (29.19)	1	-	-			
Yes	731 (41.48)	0.81	0.64, 1.02	0.07			
**Ever tested for HIV**	2830						
Yes	1161	1	-	-			
No	1669	0.55	0.46, 0.66	<0.0005			
**Received free needles and syringes in past 12 months**	1361						
Yes	1297 (35.0)	1	-	-			
No	64 (32.81)	0.75	0.43, 1.32	0.32			
**Ever been in a drug rehabilitation center?**	3005						
No	1999 (25.66)	1	-	-			
Yes	999 (41.54)	2.21	1.86, 2.64	<0.0005			
Don’t know/no response	7 (42.86)	2.39	0.53, 10.79	0.26			
**HBsAg+**	3009						
No	2554 (30.50)	1	-	-	1		
Yes	455 (33.63)	1.19	0.94, 1.49	0.14	2.09	1.01, 4.34	0.05
**total anti-HBc+**	2585						
No	785 (17.45)	1	-	-	1		
Yes	1800 (36.56)	2.40	1.93, 2.99	<0.0005	1.29	0.99, 1.68	0.06
**HCV Ag/Ab+**	3004						
No	1,224 (40.8)	1	-	-	1	-	-
Yes	1780 (59.3)	29.84	20.64, 43.16	<0.0005	19.58	19.46, 132.84	<0.0005

In adjusted analysis, which controlled for controlled for age category, duration of injecting drug use, type of drug use, current HBV infection, previous HBV infection status, and past or current HCV infection status, HIV infection was positively associated with MWID aged 24–30 years (relative to those aged 18–24 years; aOR: 1.85, 1.30–2.62), those injecting drugs longer than 3 years (relative to those injecting less than 3 years; aOR (3–5 years): 1.74, 1.04–2.93, aOR (> = 5years): 3.01, 1.87–4.89), and negatively associated with MWID who do not use heroin (aOR: 0.24, 0.08–0.68) (Table 3b). Both HBsAg+ (aOR: 2.09, 1.01–4.34) and HCV Ag/Ab+ (aOR: 19.58, 13.07–29.33) were also significantly associated with HIV infection in the adjusted analysis. While not statistically significant at the defined level, total anti-HBc+ among those HBsAg-(aOR: 1.29, 0.99–1.68) appears to indicate an association with HIV infection. No collinearity was identified among the variables included in the final adjusted analysis.

## Conclusion

This study indicates that the prevalence of HIV, HBV, HCV and their co-infection among MWID in Vietnam are high in the majority of provinces sampled. HIV infection is associated with previous HBV infection and with current or past HCV infection (HCV Ag/Ab+) in both provincial and individual-level analyses. Injection of heroin, as opposed to other drugs, is associated with HIV infection as is an intermediate age range (24–30 years of age) and longer duration of injection drug use, in-line with expectations.

Our findings support previous, but localized studies, which indicate that the burden of HIV, HBV, and HCV infection is high among MWID in Vietnam.[2,16,17,29,30] While the point prevalence of HIV at the provincial level appears to be higher than what is reported through the Vietnam annual sentinel surveillance, the wide variation by province is similar for this study and the sentinel surveillance data. This supports the geographically heterogeneous characterization [15] of the HIV epidemic among MWID that may be due to a variety of factors in each province including: key population sizes (e.g. MWID), availability of HIV/AIDS prevention and care services (e.g. coverage of needles and syringe exchange programs, HIV counseling and testing, and HIV/AIDS treatment), duration of risk (e.g. duration of injecting use), and risk behaviors (e.g. types of drugs used, frequency of needle sharing among MWID), and drug trafficking routes that often enter Vietnam from the north and north-west potentially leading to increasing injecting drug use and associated HIV. This study provides a more geographically and population-representative profile of these associations and correlations than had previously been available for MWID in Vietnam and provides both provincial-specific estimations of HIV, HCV, and HBV burden and relationship that may also be considered for the national context. Specifically, it provides previously unavailable estimation about the significant association between HIV infection and current, active HBV infection (HBsAg+) and indication of an association between HIV infection with previous infection with HBV (total anti-HBc+) when controlling for other key factors. The absolute burden of HCV and its substantial association with HIV infection further indicates a clear clinical and public health issue for MWID in Vietnam, particularly given the relative efficiency of HCV transmission via blood from needle sharing among MWID.[31]MWID

The association between HIV infection and infection with HBV and HCV when adjusted for demographics, behavior, and service uptake provide evidence that MWID in Vietnam should be counseled on risks and risk reduction for HIV, HBV, and HCV (e.g. cessation, reduction of injection drug use, non-sharing of needles and syringes, consistent condom use). Services such as opioid substitution therapy should be provided as early as possible in their injecting lifetime to prevent acquiring and transmitting HIV, HBV, and HCV. Intervening early (e.g. within 1 year of injecting drug use initiation and at an earlier age) with MWIDs may have a substantially positive impact not only on HIV infection but also on associated HBV and HCV infection or HCV re-infection. This may be relevant especially among heroin injectors who make up an estimated 85% of drug users in Vietnam.[32]

Given that Vietnam’s estimated 335,000 MWID represent the most-at-risk-population for acquiring and transmitting HIV and HBV and HCV, accurate and timely data are critical for understanding the epidemiologic profile as well as designing and expanding appropriate intervention and care programs for those at risk for such HBV/HIV and HCV/HIV. We believe that this study provides an example to the Government of Vietnam (GVN) and other stakeholders of more integrated, and potentially more efficient, disease surveillance, particularly among populations such as MWID that are at increased risk for HIV and viral hepatitis.

HIV/AIDS prevention, care, and treatment services in Vietnam have expanded considerably over the past decade, primarily through initiatives supported by PEFPAR) the Global Fund Against HIV/AIDS, TB, and Malaria, as well as projects supported by the World Bank and the UK Department for International Development. These initiatives have supported the GVN to expand HIV treatment, medical methadone therapy (MMT), and various harm-reduction efforts for MWID. The Vietnam government has set an ambitious goal to initiate 80,000 injecting drug users on MMT by 2015 although significant barriers exist towards achieving that including conflicting policies for the treatment or incarceration of PWID as well as decreasing financial support from domestic and international sources.[32] There were 22,000 PWIDs that had initiated MMT as of late 2014.[33] Despite progress in HIV/AIDS prevention and control in Vietnam, a limited number of programs and strategies focus on other key co-infections, such as HBV or HCV.[34,35] The potential impact of integrated prevention interventions are further supported by data from this study indicating that, in addition to its association with HIV infection, increased duration of injecting drug use (i.e. > = 1 year) among enrolled MWID injecting heroin is significantly associated with prior HBV infection (data not shown).

Treatment programs for HIV and HBV and HCV should be fully integrated and evaluated. In February 2011, revision to the Vietnam ART guidelines recommended the use of tenofovir (TDF) and lamivudine (3TC) containing regimens that would provide an additional antiviral effect against HBV and also potentially improve HCV-related outcomes.[36,37] Such control efforts are especially critical given a recent study from Vietnam that reports a high (15.4%) of hepatitis D virus among those with current HBV infection which may have a negative impact on HBV-related outcomes.[38] International guidelines also indicate the need for hepatitis B vaccination, screening and the appropriate care of HBV and HCV as a key component of comprehensive HIV prevention, care, and treatment among MWID [5,39–41] This will be particularly important as increased access to HIV treatment in Vietnam will likely contribute to longer life-spans of HIV-infected people, resulting in a positive impact on the chronic morbidity and mortality of HBV and HCV-associated liver disease.[10,42,43]

There are several limitations and potential biases to consider with these findings. We are unable to make inferences about the temporal relationships among injecting drug use, infection with HIV, and infection with HBV or HCV due to the cross-sectional design of the study and because we did not have sufficient serum to test for markers of acute HBV or HCV infection. However if one assumes that injecting drug use is a significant mode of infection for HIV, HBV, and HCV among MWID, then HCV and HBV infection likely precede HIV infection due to their ease of transmission relative to HIV. [39,44,45] Also because of insufficient serum, we did not test for anti-HBs, so we were unable to identify occult HBV or to define the frequency of immunity to HBV obtained through vaccination. However, given the low coverage rates of HBV vaccination in this age group and population [16], few participants in this study are likely to have been previously vaccinated. Factors associated with viral infection were analyzed with all provinces combined under the assumption that associations were similar across provinces and to increase power of the study to provide valid inferences that could more readily be translated into policy and practice. In addition, sexual orientation was not assessed in this survey which limits any analysis of male-male sex behavior as a risk factor. Such data should be considered for future research. Finally, our sampling frame, while larger than any previous survey, was not designed to be nationally representative which might limit the generalizability of these findings to all provinces in Vietnam.

In conclusion this study provides important information on the profile and burden of HIV, HBV, HCV, and their associated co-infection among MWID in Vietnam. Such estimates are important for advocating for funding and political commitment and for developing, and monitoring comprehensive, effective harm-reduction and universal vaccination programs that simultaneously address viral hepatitis and HIV/AIDS. Our results support the conclusion that adequately responding to HIV and viral hepatitis among MWID requires integrated policies, surveillance, and prevention, screening, and treatment programs.

## Supporting Information

S1 Table HIV, Viral Hepatitis, and Co-infection prevalence by province. (DOCX)
