# Supplementary material for: Findings from Integrated Behavioral and Biologic Survey among Males Who Inject Drugs (MWID) — Vietnam, 2009–2010: Evidence of the Need for an Integrated Response to HIV, Hepatitis B Virus, and Hepatitis C Virus
Source: PLoS One. 2015 Feb 18;10(2):e0118304. doi: 10.1371/journal.pone.0118304 (PMC4333571; doi:10.1371/journal.pone.0118304)
Supplement: S1 Table — (DOCX) [file pone.0118304.s001.docx]

| **Supplemental Table:** HIV, Viral Hepatitis, and Co-infection prevalence by province | | | | | | | | | | | | | | | | | | |
| --- | --- | --- | --- | --- | --- | --- | --- | --- | --- | --- | --- | --- | --- | --- | --- | --- | --- | --- |
|  | | **Province** | | | | | | | | | | | | | | | | |
| **Sero-marker** | | **Hanoi** | | | **Hai Phong** | | **Quang Ninh** | | **Nghe An** | | **Yen Bai** | | **Da Nang** | **Dong Nai** | | **HCMC** | **Can Tho** | **An Giang** |
| HIV % (95% CI) | | 14.1 (9.1, 19.0) | | | 48.0, 42.3, 53.7) | | 55.5 (49.9, 61.1) | | 23.8 (19.0, 28.7) | | 37.1 (32.0, 42.2) | | 1 (0, 2.4) | 28.7 (23.5, 33.8) | | 52.4 44.2, 60.6) | 27.6 (19.8, 35.4) | 15.7 (11.5, 19.8) |
| HBsAg+ % (95% CI) | | 17.8 (12.1, 23.5) | | | 15.4 (11.3, 19.5) | | 11.7 (8.1, 15.4) | | 12.8 (9.0, 16.5) | | 12.1 (8.6, 15.5) | | 16.7 (11.9, 21.5) | 11.7 (8.0, 15.3) | | 28.0 (18.9, 37.2) | 12.2 (7.5, 16.7) | 15.7 (11.5, 19.8) |
| HIV+ | | 15.6 | | | 17.5 | | 9.6 | | 15.5 | | 14.7 | | 66.7 | 15.1 | | 25.9 | 15.7 | 17 |
| HIV- | | 18.1 | | | 13.6 | | 14.3 | | 11.9 | | 10.5 | | 16.5 | 10.3 | | 22.8 | 13.3 | 15.4 |
| HBsAg-/total anti-HBc+ % (95% CI) | | 67.1 (58.1, 76.0) | | | 71.3 (65.7, 76.8) | | 79.4 (74.5, 84.3) | | 62.1 (56.3, 68.0) | | 79.0 (74.5, 83.5) | | 49.9 (41.8, 58.1) | 56.2 (50.2, 62.2) | | 83.1(77.6,88.5) | 77.2 (68.8, 85.7) | 71.5 (65.9, 77.0) |
| HIV+ | | 80.1 | | | 82.5 | | 89.1 | | 31.7 | | 85 | | 100 | 75.3 | | 77.6 | 82.2 | 77.5 |
| HIV- | | 52.3 | | | 61.2 | | 67 | | 68.3 | | 62.4 | | 51.1 | 49 | | 78.1 | 71.9 | 70.4 |
| HCV Ag/Ab+ % (95% CI) | | 51.0 (42.3, 59.7) | | | 75.8 (71.0, 80.7) | | 84.6 (80.5, 88.7) | | 53.0 (47.3, 58.7) | | 87.4 (83.9, 90.9) | | 10.9 (7.3, 14.5) | 41.9 (36.3, 47.6) | | 71.0 (63.6, 78.4) | 54.6 (45.2, 64.1) | 44.7 (39.0, 50.3) |
| HIV+ | | 35.5 | | | 93.7 | | 99.4 | | 95.8 | | 98.5 | | ** | 89.5 | | 97.2 | 98.8 | 91.5 |
| HIV- | | 64.5 | | | 59.4 | | 66.2 | | 39.7 | | 80.8 | | 10.9 | 22.6 | | 44.9 | 69.4 | 36 |
| **Hepatitis and HIV prevalence (among all enrollees with a recorded result)** | | | | | | | | | | | | | | | | | | |
| HBsAg+/HIV+ % (95% CI) | | 2.2  (0.3, 4.0) | | | 8.4  (5.2, 11.5) | | 5.4  (2.8, 7.9) | | 3.7  (1.5, 5.8) | | 5.5  (3.1, 7.9) | | 0.9  (0, 2.3) | 4.3  (2.0, 6.6) | | 13.4  (7.5, 19.4) | 4.9  (1.9, 7.8) | 2.7  (0.8, 4.5) |
| anti-HBc+/HIV+ % (95% CI) | | 13.4 (7.7, 19.2) | | | 39.0 (33.0, 45.0) | | 50 (43.9, 56.1) | | 19.7 (14.9, 24.5) | | 32.0 (26.8 (37.1) | | 0.1 (0, 0.2) | 20.8 (15.9, 25.6) | | 38.7 (29.9, 47.4) | 18.7 (12.0, 25.5) | 12.1 (8.1, 16.1) |
| HCV Ag/Ab+/HIV+ % (95% CI) | | 14.1 | | | 50 | | 55.2 | | 22.8 | | 36.5 | |  | 25.8 | | 46.4 | 26.8 | 14.3 |
| HBsAg+/HCV Ag/Ab+/HIV+ % (95% CI) | | 0.3 (0, 0.8) | | | 1.2 (0.2, 3.2) | | ** | | 1.7 (0.2, 3.2) | | 2.9 (1.1, 4.8) | | ** | ** | | ** | ** | 0.3 (0, 1.0) |
| * indicate p-value <0.005 | | ** no observations | | |  | |  | |  | |  | |  |  | |  |  |  |
|  |  | |  |  | |  | |  | |  | |  | | |  |  |  |  |
